# Supplementary material for: Amniotic LPS-Induced Apoptosis in the Fetal Brain Is Suppressed by Vaginal LPS Preconditioning but Is Promoted by Continuous Ischemic Reperfusion
Source: Int J Mol Sci. 2022 Feb 4;23(3):1787. doi: 10.3390/ijms23031787 (PMC8836254; doi:10.3390/ijms23031787)
Supplement: Supplementary file 1 [file ijms-23-01787-s001.zip › ijms-1510365-supplementary.pdf]

Supplementary Figure S1

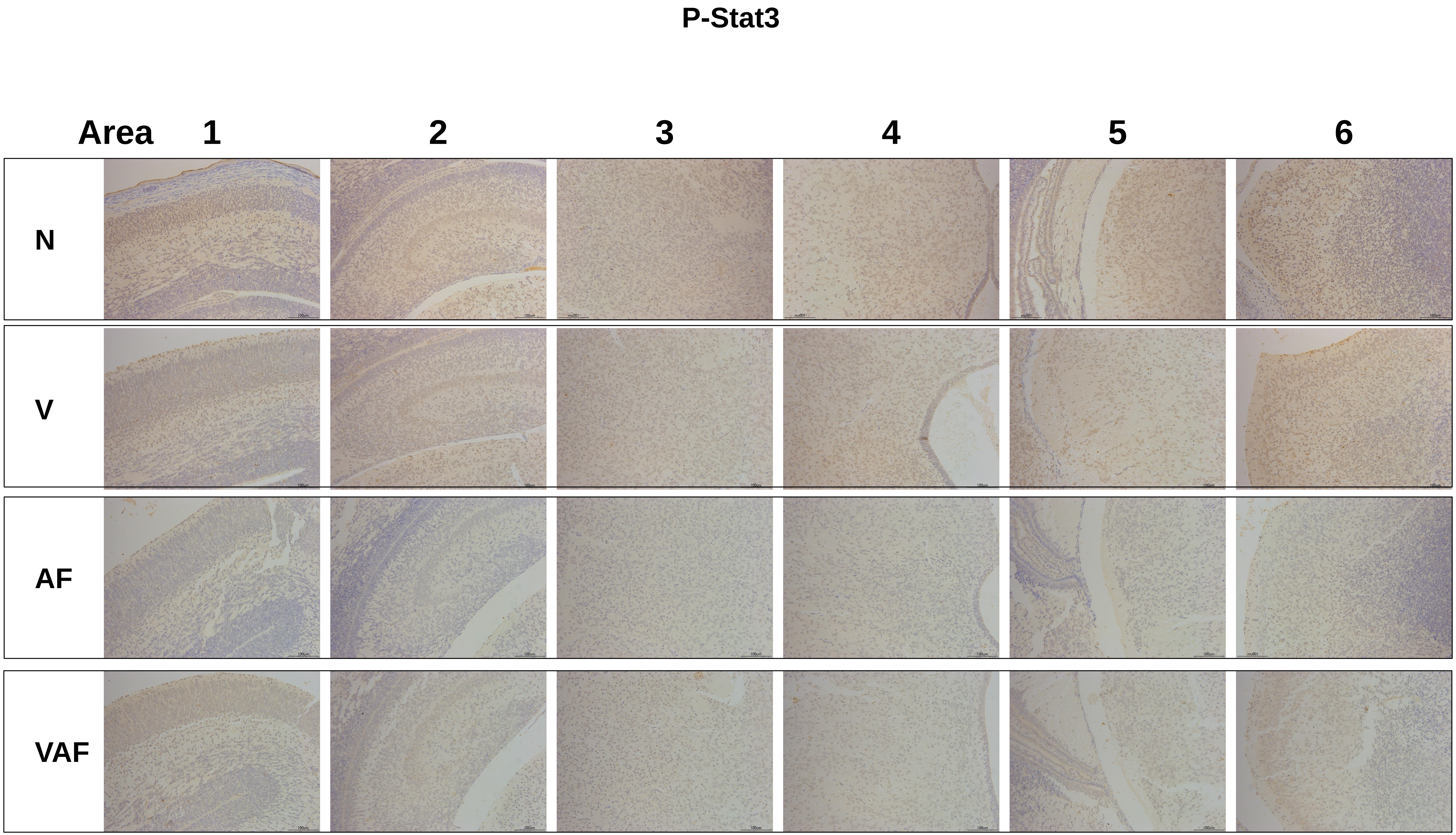

Supplementary Figure S2

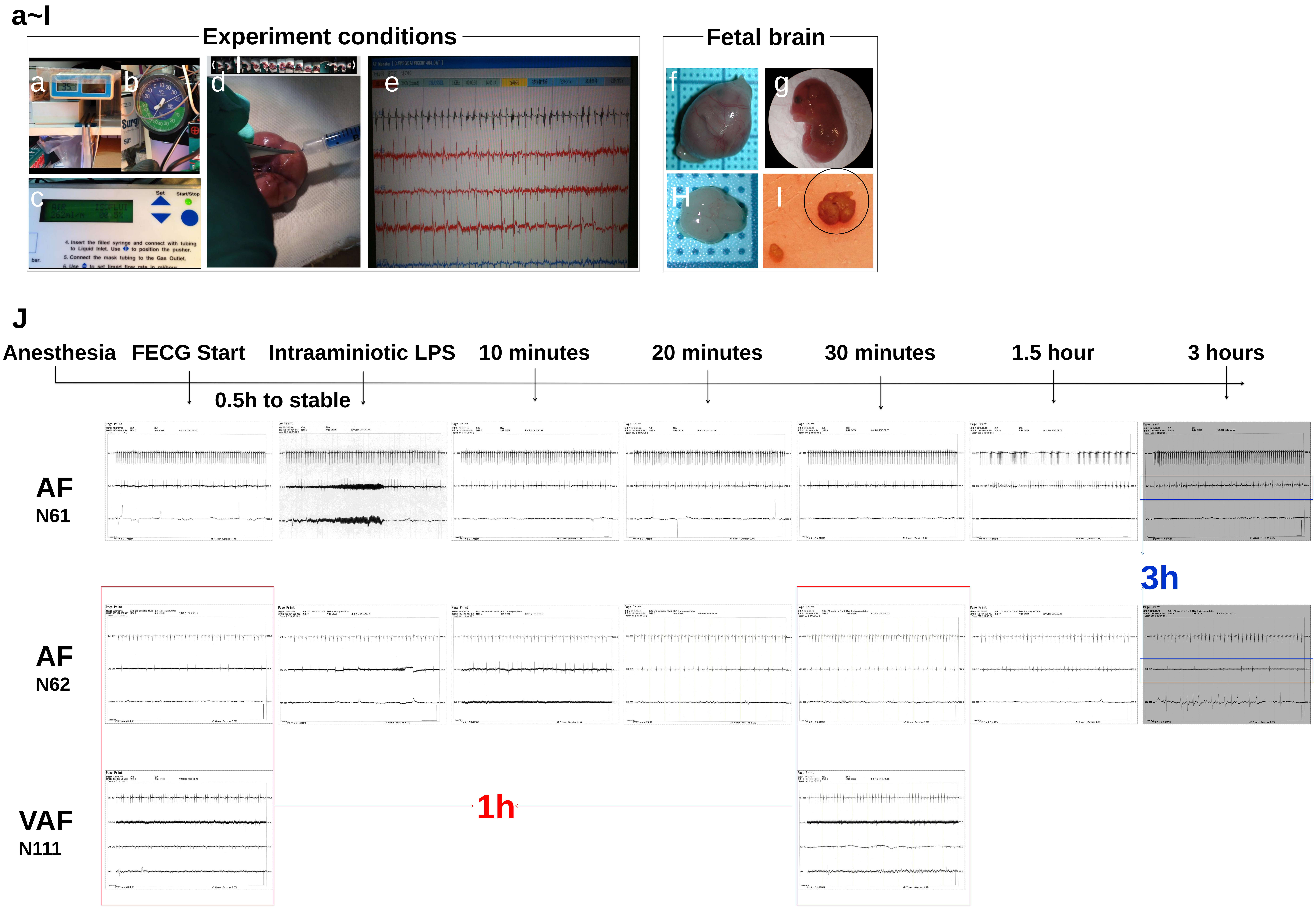

Supplementary Figure S3. All primers used in this study.

mGM-csf_F444	aagaggtagaagtcgtctctaacgag
mGM-csf_R593	agtatgtctggtagtagctggctgt
mTLR4_F1942	gtggtatccactgtagcatttctg
mTLR4_R2149	agtctctgtagtgaaggcagaggt
mTLR3_F2188	ccagacatctctaaggtcactgaa
mTLR3_R2399	tgttacagaggtagtgagtggacag
mTLR2_F810	ccctcaggataggaaatgtagagac
mTLR2_R976	gcttaagtgaagagtcaggtgatg
mG-csf_F706	agcagaaagccctttccagatagt
mG-csf_R872	aagagccttctctctctgctctaag
IL-6_F385_F399	tcttctggagtaccatagctacctg
IL-6_R580_R546	ctgtgactccagcttatctgttagg
TNFa_1F	gatttgctatctcataccaggagaa
TNFa_1R	acagagcaatgactccaaagtagac
IL1b_1F	aaacggtttgtcttcaacaagatag
IL1b_1R	attccatggtgaagtcaattatgtc

Supplementary Figure 4. All antibodies used in this study.

Name	Cat. No.	MW	Dilution folds Imaging experient	Isotype		Dilution folds W.B
CreB	9197	43	6,400x	Rabbit	IgG	1000x
P-CREB 488	9187	43	200x	Rabbit	IgG	
P-CREB	9191	43		Rabbit	IgG	1000x
JNK	9258	46/54		Rabbit	IgG	1000x
p-JNK	4668	46/54	50x	Rabbit	IgG	1000x
p38	9212	43		Rabbit		1000x
p-p38	9211	43	200x	Rabbit		1000x
ERK1/2	9102	44/42	250x	Rabbit	IgG	1000x
p-ERK1/2	4376	44/42	400x	Rabbit		1000x
Stat3	9139	79/86		Mouse	IgG2a	1000x
P-stat3	9131	79/86	100x	Rabbit		1000x
NF-kB	4717	120		Rabbit		1000x
MAP2	ab28032	200	1000x	Mouse	IgG1	1000x
p-S6	2211	32		Rabbit		1000x
DJ-1	2134	22		Rabbit	IgG	1000x
Neurofilament L	2835	70	50x	Mouse	IgG1	1000x
LC3a/B	2775	16/14	1000x	Rabbit	IgG	1000x
alpha-synuclein	4179	18	200x	Rabbit	IgG	1000x
beta-actin	5125	45	200x	Rabbit	IgG	1000x
ATF2	9221	70		Rabbit	IgG	1000x
